# Using intervention mapping for hookah smoking cessation: a quasi-experimental evaluation

**DOI:** 10.1186/s13722-022-00287-5

**Published:** 2022-03-14

**Authors:** Sakineh Dadipoor, Ali Heyrani, Mehdi Mirzaei-Alavijeh, Teamur Aghamolaei, Mohtasham Ghaffari, Amin Ghanbarnejad

**Affiliations:** 1 Tobacco and Health Research Center, Hormozgan University of Medical Sciences, Bandar Abbas, Iran; 2 Social Determinants in Health Promotion Research Center, Hormozgan Health Institute, Hormozgan University of Medical Sciences, Bandar Abbas, Iran; 3 Social Development and Health Promotion Research Center, Health Institute, Kermanshah University of Medical Sciences, Kermanshah, Iran; 4 Cardiovascular Research Center, Hormozgan University of Medical Sciences, Bandar Abbas, Iran; 5 Environmental and Occupational Hazards Control Research Center, School of Public Health & Safety, Shahid Beheshti University of Medical Sciences, Tehran, Islamic Republic of Iran; 6 Department of Epidemiology And Biostatistics, School of Public Health, Tehran University of Medical Sciences, Tehran, Iran

**Keywords:** Experiment, Hookah smoking, Intervention mapping, Tobacco, Women

## Abstract

**Background:**

The present study aimed to evaluate the results of a theory-based and systematic intervention on Hookah Tobacco Smoking (HTS) cessation in women local to Bandar Abbas, Iran.

**Methods:**

In the present quasi-experimental research, we used an intervention mapping approach to develop, implement, and evaluate an education and training course as our intervention. Applying the results of a systematic review and two prior local qualitative studies, we identified six HTS determinants and set goals for the intervention. We selected 212 eligible women through systematic stratified random sampling and enrolled them in control and intervention groups. The course was presented to the intervention group in 17 sessions for four months. The educational material was developed to address the goals of the intervention, improve HTS determinants, and change the HTS behavior. We used a questionnaire to collect data on participants’ characteristics, HTS behavior, and detailed determinants of HTS in the control and intervention groups at the beginning of the study, at the end of the intervention, and at three- and six- months follow-up. All work done in the study was guided by ethical considerations.

**Results:**

The results showed no significant difference between women enrolled in control and intervention groups regarding participants’ characteristics and HTS behavior. At baseline, there were no differences between groups for six determinants of HTS (knowledge, attitude, social norms, self-efficacy, habit, and intention). At the end of the intervention and at three and six months follow-up, the women in the intervention group had significantly better results in all six domains, compared with those in the control group. The rate of HTS abstinence at the end of the intervention and at the three- and six-month follow-ups was 61.3%, 48.5%, and 45.5% for the intervention and 16%, 14.4%, and 10% for the control groups, respectively.

**Conclusions:**

HTS is a complicated behavior, and its cessation is hard. However, Intervention Mapping (IM) can be a powerful integrative, purposeful, theory-based, and participation-based method to reduce or cease HTS. This method should be tested in other settings.

*Trial registration*: IRCT20190126042494N1, Registered 3.3.2019. https://en.irct.ir/trial/37129

**Supplementary Information:**

The online version contains supplementary material available at 10.1186/s13722-022-00287-5.

## Introduction

Hookah is a device used for smoking tobacco. Hookah Tobacco Smoking (HTS) is considered a growing health issue as it is accompanied by disease, disability, and addiction in some consumers [1]. The rate of HTS is increasing worldwide. In 2018, a review article reported HTS in 37.2% of East Mediterranean, 22.7% of European, and 11.4% of American adults [2]. Because of greater perceived social acceptance and positive attitudes, HTS is more common in women [3–7]. Iran is among the countries with a significant rise in hookah tobacco consumption. [8, 9]. In the southern Hormozgan Province, 28.4% of men and 45.2% of women smoked hookahs in 2011 [10].

HTS is related to a higher risk of various medical conditions, including premature menopause, low bone density, infertility, ectopic pregnancy, infantile disease and mortality, intrauterine growth restriction, increased chromosome disorders, and genital warts in women [11–13]. Considering the high prevalence of HTS among women and its adverse effects, it is essential to identify effective interventions to address HTS. HTS cessation can benefit from effective and relevant behavior change techniques. Yet, it has been shown that the low rate of participation in interventional behavior change programs in adult smokers is a key barrier [14].

Due to the complexity of tobacco consumption behavior and its association with socio-structural processes, certain models and theories have been proposed to tackle the issue [15]. Using a systematic, integrated, participatory, and theory-based approach that benefits from effective, relevant behavior change techniques is essential to for HTS cessation. Intervention Mapping (IM) introduces a series of steps and procedures to help health promoters design evidence- and theory-based programs. It describes the planning process of health promotion in six steps: 1. needs assessment 2. creating the matrix of goals for behavior change 3. choosing theory-based approaches and practical strategies 4. developing interventions 5. planning the implementation of program 6. planning the evaluation [16]. In other studies, IM has been used successfully to determine and implement behavioral and environmental interventions in cigarette smoking and substance use control [17–19].

To the researchers’ knowledge, fewer studies have focused on HTS behavior control. The majority of research has addressed the critical factors involved in HTS [20–23]. The other related literature on HTS among women focused on such aspects as awareness of and attitudes towards HTS [24], the prevalence of HTS [8, 24], factors underlying HTS [25], and the correlation of HTS and health outcomes [26]. fewer studies on HTS cessation have been conducted among men (excluding women) without a precise needs assessment and participation of the target group in interventions [27, 28]. This research, carried out in Bandar Abbas (Hormozgan Province, in the south of Iran), is pioneering in the use of the IM framework to design, implement, and evaluate HTS cessation among women.

## Methods

### Design

**Fig. 1:**
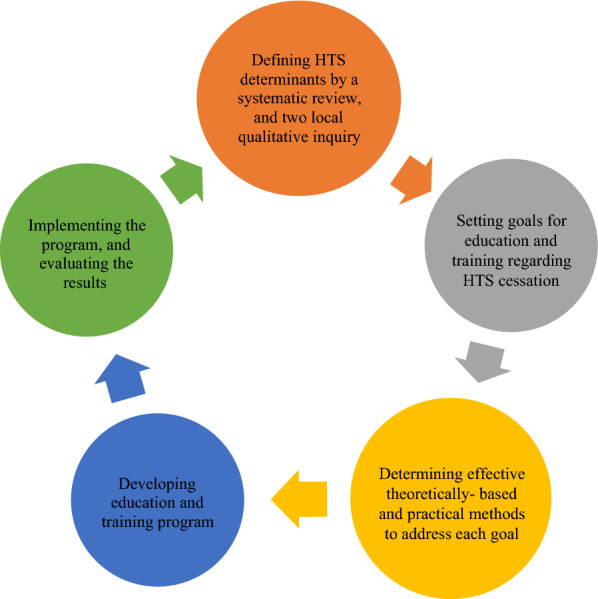
Steps of intervention (Education and Training for HTS Cessation) design based on IM

This quasi-experimental study was conducted between June 2018 and July 2020 on intervention and control groups of women over 15 years of age living in Bandar Abbas. The intervention, namely education and training for HTS cessation, was designed and implemented in five systematic steps according to the IM framework (Fig. 1). In the first step, the results of a systematic review and two local qualitative studies, previously done and published elsewhere [29–31], were used to better understand the behavioral and environmental factors associated with HTS among women. The details of the other steps can be found in the published research protocol [32].

### Participants’ eligibility

The participants were women over 15 years of age. The inclusion criteria were smoking hookah tobacco four times a week for at least six months [33, 34], no prior experience of a psychological disorder or cardiovascular diseases [35], being local to Bandar Abbas City or residing in the city for at least ten years (for cultural adjustment), and signing an informed consent to take part in the research. The exclusion criteria were simultaneous smoking of cigarettes, tobacco products or drugs, attempts to cease HTS at the outset of the study or just before the study, using nicotine alternative therapy, participation in any other cessation program, absence from more than two training sessions, and unavailability in the post-test.

### Sample size and sampling

We used the standard sample size formula in comparative studies with d = 1, s1 = 2.55, and s2 = 2.38. Similar research findings were consulted measuring self-efficacy three months after training in the intervention and control groups by Sotoude [36]. The other values were set at α = 0.05, β = 0.2, a power of 80% and 5% attrition. The sample size for each group was estimated at 106.

Four healthcare centers were selected randomly from 20 centers serving Bandar Abbas. Two were assigned for the control group and the other two for the intervention group. In each healthcare center, an alphabetic list of women over 15 years old was obtained. One of the women was then selected randomly. We called her on the phone to determine study eligibility. If she was found to be ineligible, the next woman on the list was contacted. This process continued until an eligible participant was identified. The next eligible participants were searched for by adding 53 to the row number of the previously selected woman and proceeding on the abovementioned process. We selected 53 eligible women in each healthcare center to be included in the study, totaling 106 for the control group, and the same for the intervention group.

### Measurement

The instrument was developed based on the first two steps of the IM framework in our study [37]. It was a questionnaire composed of two parts. The first part asked about demographic and HTS behavioral information, including age, education level, marital status, job status, socio-economic status, the age at which HTS began, years of HTS, HTS in family members, and frequency of HTS per week. The second part asked about detailed determinants of HTS behavior (Table 1).

**Table 1:** Description of the research instrument

Determinants	No. of Items (Format) in the questionnaire	Scoring (Range)	Internal consistency (Cronbach's alpha)	Item Example
(1) Knowledge	Ten items (Multiple Choice Questions)	True/ False/ Don't know	0.79	The smoke is purified in the water tank of hookah, so it does not have any harm
(2) Attitudes	15 items (Likert Scale Questions)	Strongly Disagree = 1, Disagree = 2, No idea = 3, Agree = 4, Strongly Agree = 5	0.84	HTS is dangerous to my health
(3) Social Norms	20 items (Likert Scale Questions)	Absolutely important = 1, Important = 2, No idea = 3, Unimportant = 4, Absolutely unimportant = 5,	0.85	My family and friends expect me to replace a healthier behavior instead of HTS
(4) Self-efficacy	Nine items (Rating Scale Question)	The least (1) to the most (10)	0.78	To what extent are you sure about breaking up with HTS friends?
(5) Habit	Seven items (Likert Scale Questions)	Strongly Disagree = 1, Disagree = 2, No idea = 3, Agree = 4, Strongly Agree = 5	0.86	As I have smoked hookahs for years, I cannot quit it
(6) Intention	1 Item (Dichotomous Question)	Yes/ No	0.80	Do you intend to cease HTS now?
(7) behavior	1 Item (Numeric Text Question)	Number of HTS sessions per week		How often do you smoke hookah in a week?

### Data collection

The data were collected by questionnaires completed as written self-reports by participants before and after the intervention. After signing informed consent, all control and intervention participants were given the pre-test questionnaire just before the intervention (i.e., at the beginning of the first session). The intervention group then gave the post-tests on three occasions: at the end of intervention (i.e., the 14^th^ session) and at three- and six-month follow-ups. The control group was also given questionnaires at the same time as the intervention group. For all illiterate participants, the questions were read by the interviewer (SD) to eliminate any bias. Each questionnaire took an average of 25 min to complete.

### Intervention delivery and follow-up

The intervention was a participatory education and training course designed at personal and interpersonal levels. IM recommends interventions be conducted in five personal, interpersonal, organizational, social, and political levels for maximum effectiveness. Yet, we confined the intervention to two levels due to time and coordination limitations.

We held 14 sessions in intervention groups of 10 to 15 women enrolled randomly at the personal level. At the interpersonal level, three sessions were held for people who had been introduced by the participating women in the intervention group as their supporters. SD enlisted these people, contacted them by phone, explained the purpose and process of intervention, and obtained their informed consent to participate. These people were also organized in groups of 15 to 20 randomly. The control group attended a one-hour session of instructions on the detriments of HTS in six groups of 15 to 20.

The entire course took four months, from September 2019 to January 2020. All sessions were conducted through short, challenging lectures followed by participants’ guided discussions. According to the intervention program, the participants did not receive any other relevant education or training during the intervention.

Four people presented the course. An expert in health education and promotion with a good knowledge of HTS behavior (SD) guided nine sessions. A physician and a clinical psychologist, both experienced in managing physical and psychological consequences of tobacco cessation, conducted the other five sessions. A competent woman who had successfully stopped HTS acted in all sessions as a role model. Sheimproved women’s self-confidence and self-efficacy, and shared her experience in hookah cessation. The instructional material is appended in Additional file 1.

**Fig. 2:**
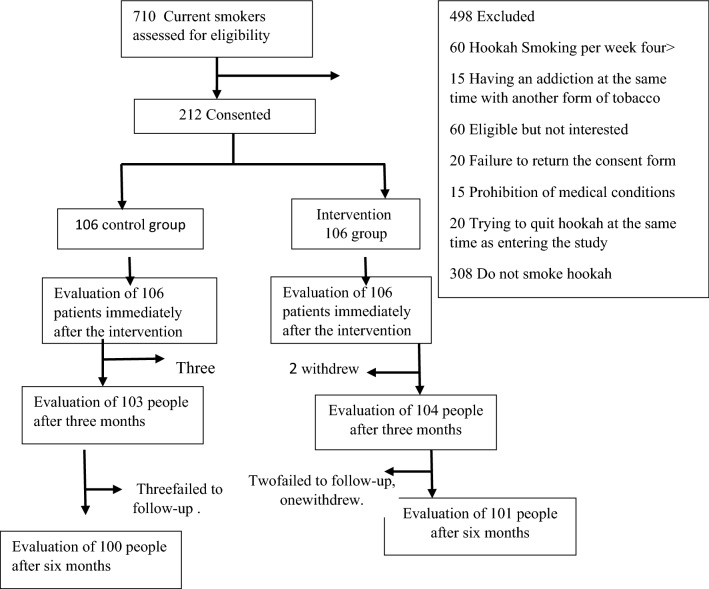
Intervention profile

The time and place (e.g., mosque, neighborhood council public hall, or healthcare center) of each session was agreed upon by participants. Each session lasted 90 to 180 min, with a 10-min break after each 40-min discussion. SD attempted to lower the attrition rate by calling the participants on a regular weekly basis. However, three women from the control group and two from the intervention group withdrew at the end of the course. We failed to follow three women from the control and two from the intervention group at the three-month follow-up. One more woman from the intervention group withdrew at the three-month follow-up (Fig. 2).

### Intervention outputs

The intended outputs of the intervention included an increased awareness about harms of HTS and the benefits of cessation, a positive attitude toward HTS cessation, an appreciation of and adherence to positive social norms regarding HTS cessation, an increased self-efficacy in HTS cessation, and an increased behavioral intention for HTS cessation in participants. The questionnaire measured these items at the end of the intervention and at three- and six-month follow-up.

### Intervention outcomes

#### Primary outcome

The intended primary outcome of the intervention was to help the participating women quit HTS. It was measured by asking a question besides the questionnaire immediately after the end of the intervention and at three and six months’ follow-up. The question was, "Did you manage to cease HTS?”, and the answer was *yes/ no*.

#### Secondary outcome

The secondary outcome was an eliminated habitual act of HTS investigated by asking the participants “How often do you smoke hookahs during the week?” in the questionnaire just after the end of the intervention and in three and six months’ follow-up. Then each answer was compared with the previous one.

### Data analysis

Demographic variables and HTS behavioral information of the two research groups (control and intervention) were analyzed by calculating the mean and standard deviation of the data collected by the first part of the questionnaire at the beginning of the study. Simultaneously, we used the chi-square test to search for any significant difference between the two groups. To compare the detailed determinants of HTS behavior, we analyzed the data collected in the second part of the questionnaire through independent-sample *T*-test at the beginning of the intervention, at the end of the intervention, and in three- and six-month follow-up. Moreover, we traced changes in the intended outputs and outcomes of the intervention in each group through repeated measures’ analysis at the scheduled occasions mentioned above.

## Results

Using descriptive statistics, we explored the demographic characteristics and HTS behavioral information of the two research groups (Table 2). Moreover, the results showed no significant difference between the women enrolled in control and intervention groups regarding age (p-value = 0.75), education level (p-value = 0.581), marital status (p-value = 0.708), job status (p-valye = 0.726), socio-economic status (p-value = 0.999), the age at which HTS began (p-value = 0.835), years of HTS (p-value = 0.10), HTS in family members (p-value = 0.157), and frequency of HTS per week (p-value = 0.496).

**Table 2:** Demographic characteristics and HTS behavioral variables in the control and the intervention group

Variable	Total sample (N = 212)	Intervention group (n = 106)	Control group (n = 106)	p-value
Age (M,SD)	37.7 (13.8)	39.4 (14.4)	35.9 (12.9)	0.75
Educational level				
Illiterate	29 (13.7%)	17 (58.6%)	12 (41.4%)	0.581
Primary	46 (21.7%)	25 (54.3%)	21 (45.7%)	
Secondary	56 (26.4%)	25 (44.6%)	31 (55.4%)	
Diploma	58 (27.4%)	26 (44.8%)	32 (55.2%)	
College	23 (10.8%)	13 (56.5%)	10 (43.5%)	
Marital status				
Never married	34 (16%)	16 (15.1%)	18 (17.0%)	0.708
Ever married	178 (84%)	90 (84.9%)	88 (83%)	
Job-status				
Not working	172 (81.1%)	87 (82.1%)	85 (80.2%)	0.726
Working outside home	40 (18.9%)	19 (17.9%)	21 (19.8%)	
Socio-economic status				
Low	34 (16%)	17 (16%)	17 (16%)	0.999
Middle	108 (50.9%)	54 (50.9%)	54 (50.9%)	
High	70 (33%)	35 (33)	35 (33%)	
HTS initiation age				
< 15	45 (21.2%)	24 (22.6%)	21 (19.8%)	0.835
15–30	148 (69.8%)	72 (67.9%)	76 (71.7%)	
> 30	19 (9.0%)	10 (9.4%)	9 (8.5%)	
Years of HTS				
< 5	62 (29.2%)	28 (26.4%)	34 (32.1%)	0.10
5–15	61 (28.8%)	23 (21.7%)	38 (35.8%)	
< 15	89 (42.0%)	55 (51.9%)	34 (32.1%)	
HTS in Family				
Yes	132 (62.3%)	61 (57.5%)	71 (67%)	0.157
No	80 (37.7%)	45 (42.5%)	35 (33%)	
Frequency of HTS per week				
4–5	31 (14.6%)	14 (45.2%)	17 (54.8%)	0.496
6–16	60 (28.3%)	35 (58.3%)	25 (41.7%)	
17–27	50 (23.6%)	24 (48.0%)	26 (52.0%)	
> 27	71 (33.5%)	33 (46.5%)	38 (53.5%)	

Regarding intervention outputs, all six determinants of hookah tobacco smoking, namely knowledge, attitude, social norms, self-efficacy, habit, and intention, had no statistically significant difference between groups at the beginning of the study. Then after the intervention and during the follow-up, the results showed a statistically significant between-group difference (all with a p-value of 0.001). The intervention group obtained significantly better results in all six domains at the end of the intervention and in the three- and six-months follow-up (all with a p-value less than 0.001 in repeated measures analysis). At the follow-up points, the women in the control group did not show a significant improvement in the determinants except in knowledge and attitude (Table 3).

**Table 3:** Determinants of HTS at the particular occasions during the study in control and the intervention groups

Variables	Groups	Before intervention (Mean ± SD)	After intervention (Mean ± SD)	Three months after intervention (Mean ± SD)	Six months after the intervention (Mean ± SD)	P-value*
Knowledge	Intervention	4.67 ± 2.70	8.99 ± 1.08	9.06 ± 1.04	9.30 ± 0.93	< 0.001
	Control	5.00 ± 2.59	6.73 ± 1.70	7.37 ± 1.50	7.81 ± 1.42	< 0.001
	P-value	0.336	0.001	0.001	0.001	
Attitude	Intervention	46.35 ± 10.06	61.19 ± 6.13	60.00 ± 7.09	61.85 ± 11.35	< 0.001
	Control	45.29 ± 9.89	46.70 ± 10.98	48.98 ± 9.60	48.04 ± 11.44	0.003
	P-value**	0.438	0.001	0.001	0.001	
Social Norms	Intervention	62.49 ± 9.97	73.95 ± 16.29	71.63 ± 15.10	73.92 ± 18.11	< 0.001
	Control	60.83 ± 18.81	58.47 ± 18.06	60.97 ± 17.48	59.31 ± 19.86	0.254
	P-value**	0.505	0.001	0.001	0.001	
Self-efficacy	Intervention	38.16 ± 19.21	63.15 ± 11.63	62.82 ± 11.32	64.03 ± 13.27	< 0.001
	Control	41.24 ± 18.44	42.77 ± 16.73	42.21 ± 15.33	41.45 ± 12.05	0.173
	P-value**	0.236	0.001	0.001	0.001	
Habit	Intervention	25.24 ± 6.88	24.63 ± 6.43	17.19 ± 7.54	15.88 ± 9.16	< 0.001
	Control	24.63 ± 5.94 ±	27.70 ± 5.28	28.77 ± 5.34	31.99 ± 6.23	< 0.001
	P-value**	0.482	0.001	0.001	0.001	
Intention	Intervention	10.79 ± 4.77	15.62 ± 2.66	15.82 ± 2.42	16.27 ± 3.34	< 0.001
	Control	11.23 ± 4.47	12.32 ± 4.33	11.00 ± 4.62	12.45 ± 4.82	0.380
	P-value**	0.486	0.001	0.001	0.001	
Behavior***	Intervention	21.33 ± 15.41	2.84 ± 6.24	4.13 ± 7.11	3.82 ± 6.43	< 0.001
	Control	22.54 ± 18.99	17.47 ± 14.73	19.40 ± 16.62	20.23 ± 16.37	0.367
	P-value**	0.565	0.001	0.001	0.001	

^*^p-value is calculated in repeated measures analysis mode for each group (intervention, control)

^**^p-value is calculated in cross-sectional independent analysis mode between intervention and control group

^***^Frequency of HTS per week, as the secondary outcome of the intervention

**Fig. 3:**
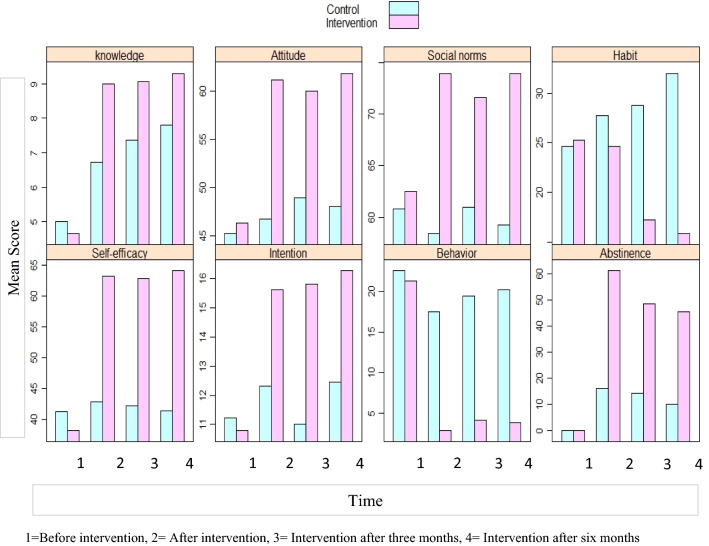
Mean HTS determinants and behavior scores (frequency of HTS per week and the cessation rate) in research groups

The trend of change in the mean scores of the determinants and HTS behavior is indicated in Fig. 3. As observed, a statistically significant change occurred in the intervention group after the intervention compared to the pre-test. The trend of change in the determinants seems to be fixed in the control group. Moreover, the mean frequency of weekly HTS in the intervention group showed to be significantly lower than the control, and the rate of HTS cessation was higher.

**Fig. 4:**
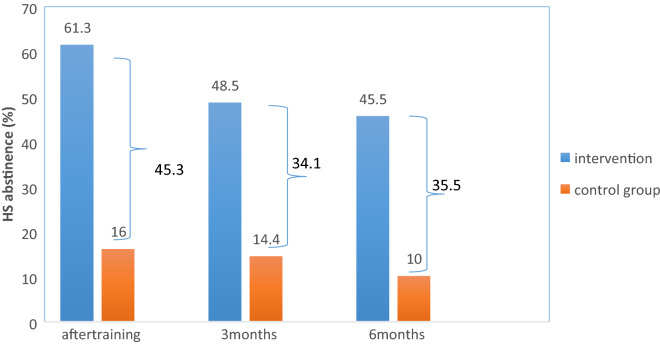
HTS abstinence at the particular occasions during the study in control and the intervention groups

The intended primary outcome of the intervention, as stated earlier, was HTS cessation. Among women in the intervention group, 61.3% ceased HTS at the end of the intervention. The rate was 16% for women in the control group. The rates of HTS cessation at the three- and six-month follow-ups were 48.5% and 45.5% for the intervention and 14.4% and 10% for the control group, respectively (Fig. 4).

The intended secondary outcome was an eliminated habitual act of HTS. The behavior showed no significant between-group difference at the beginning of the study (p-value = 0.565). During the study, the act of HTS eliminated significantly in the intervention group (p-value < 0.001) while it did not change in the control group (p-value = 0.367) (Table 3).

## Discussion

This study aimed to explore the effect of an educational intervention based on an intervention mapping of HTS cessation in women over 15 years of age in Bandar Abbas City in Hormozgan. The results showed that knowledge scores were significantly increased in the control and intervention groups after the intervention. Yet, this increase was higher in the intervention group than the control. Similarly, in some other studies, there was an increase in the knowledge (awareness) of the adverse effects of HTS in the intervention group [38, 39]. As for the increased knowledge in the control group, it may be argued that one session of educational intervention and completing the questionnaire three times increased their knowledge. It can be speculated that education increased their level of knowledge and, thus, may have motivated participants in the control group to take the first step to lower the rate of hookah smoking or cessation.

As the present findings show, the training intervention significantly changed participants’ attitudes about HTS. Women in the intervention group developed a positive attitude toward HTS cessation and a negative attitude toward HTS. This change is likely because the women developed a thorough understanding of the detriments of HTS and the benefits of cessation. Theyrecurrently reminded themselves of these detriments and benefits during and after the intervention. People's beliefs and attitudes play a key role in HTS. Generally, the more people view the outcomes as positive, the more they tend toward it [40]. In a body of research, including Momenabadi et al. and Firoozabadi et al., a training intervention based on the BASNEF model and the theory of planned behavior increased the attitude score in the intervention group [41, 42].

The present findings show that the mean HTS habit score was decreased in the intervention group after the training as compared with the control group. It is noteworthy that the farther we went from the intervention, the lower the participants’ dependence on the unhealthy habit in the intervention group. This difference was statistically significant in the 3- and 6-month follow-up. This would point to the fact that HTS habit is developed through time. Accordingly, its elimination is time-consuming. Quitting a habit can be a crucial variable in the success of HTS cessation. However, in the present research, this variable and its significance were emphasized all throughout the intervention. Yet, it seems to be inadequate and requires more time and more complicated interventions. Certainly, many internal and external variables are involved in forming a new habit. It seems that recognizing all these variables and intervening in them can help reduce or eliminate HTS. Some research revealed that people who do not develop the HTS habit could successfully cease tobacco consumption [43].

The present findings showed that the mean self-efficacy score in HTS cessation was increased in the intervention group after the training intervention as compared with the control. This divergence was statistically significant. It seems that a training intervention using effective theoretical methods and appropriate practical methods, including role models, role play, and guided practice, managed to gain women's trust and increase their confidence in their ability to cease HTS, resist the current conditions, and break from hookah smoking friends. This would point to the fact that those with a higher level of self-efficacy enjoy a more realistic view, engage more in the new behavior, and are less influenced by peer pressure [44, 45]. Previous research findings showed that the more people rely on their own capability of self-care (abstinence form tobacco consumption), the less they tend to show the target behavior [46, 47]. To explain this finding, it can be noted that those with a high level of self-efficacy are well-coordinated and stable in behavior. Thus, they are more psychologically fit. And, there are fewer chances of retreating to uncommon or unhealthy behaviors like HTS to solve problems. A body of research has confirmed the positive effect of the training intervention on increasing self-efficacy in the intervention group compared with the control group in showing HTS behavior [27, 28].

Furthermore, the present findings showed a significant increase in the mean subjective norm score in the intervention group compared with the control. It seems that influential people's recommendation in participants' lives was a key factor in ceasing or cutting down on HTS. Peers and family members play a key role in HTS and can effectively persuade people not to smoke and prevent the rate of HTS [48]. Similarly, a body of research found an increase in the mean score of subjective norms in the intervention group compared with the control [41, 49]. To confirm this finding, another study showed that family members' comments play a key role in HTS cessation [50]. Thus, it is essential to identify the influential people in smokers' lives, raise their awareness of tobacco products' detriments and involve them directly and indirectly in training interventions.

Another finding was the increased behavioral intention in the intervention group compared with the control group. The increased intention of cessation and the lower rate of HTS after the training intervention have been confirmed in a body of literature [41, 51]. In the present research, not all participants who intended to cease HTS ultimately managed to do so. It seems that there were specific barriers to changing this intention to an actual cessation behavior. In other words, other facilitating factors were needed too. These factors act as moderators to change intention to behavior and, when lacking, they disrupt the procedure [52]. Further research is required to increase the rate of HTS cessation as it mainly relates to these barriers.

The present findings showed a significant decrease in the frequency of HTS in the intervention group after the training compared with the control. This would show that the systematic and participation-based IM framework managed to affect changing HTS behavior positively. Similar to the present findings, other studies showed a statistically significant decrease in HTS in the intervention group compared with the control group [27, 28]. Contrary to the present findings, some other research showed no effect of the training intervention on HTS behavior [14, 38]. These contradictory findings can be partially explained by different training interventions, the duration of intervention, and features of the target population.

Another main finding was HTS cessation in the intervention group compared with the control group. The difference between the two groups was statistically significant. It seems that due to the complexity of the effective factors involved in HTS or cessation, IM can be a suitable method to achieve HTS cessation. Similar to the present findings, another study reported a two-fold rate of cessation in the intervention group compared with the control [53]. Contrary to the present findings, no statistically significant difference was found in some other investigations between the intervention and control groups regarding the rate of hookah cessation [54, 55]. This divergence can be partly due to the type of training intervention, duration of intervention, sample size, and characteristics of the target population. Why, in the same training conditions and the same demographic information, some women managed to cease HTS while others did not can be explained by the fact that besides intrapersonal factors, several external factors might have been at work. These factors were not controllable by the participants or researchers.

## Limitations

The data about hookah tobacco smoking (HTS) were self-reported in this study. It is possible that the women participants provided socially desirable responses. So the researcher attempted to emphasize the confidentiality of the information to lower the bias. Convenience sampling among women residents of a city in Hormozgan Province probably limits the generalizability of findings to women in other provinces and other target populations, especially men. However, to increase generalizability, the sampling was done in a big city within the province. The women were selected from different socio-demographic backgrounds.

Another limitation of this research was the lack of biochemical tests to confirm cessation. Instead, from the beginning, researchers attempted to gain the participants’ trust to reduce the effect of this bias as much as possible. Some other related literature also drew attention to the effective role of gaining participants' trust to increase their honesty in response to questions exploring their state of HTS after the training intervention [14, 56]. As there is no similar research to the present work, comparability and decision-making with this respect are difficult. Due to time limitations, any intervention at organizational, social, and political levels was deemed impossible. It is suggested that all these levels be investigated for a successful HTS cessation.

## Implications

No effective hookah cessation programs were available before the present study to intervene in HTS among men and women or any other target population. Thus, the present study significantly contributes to the HTS cessation literature. Implications of this quasi-experimental research show that women smokers who participated in the intervention had a higher cessation rate than those in the control group. This would point to the current program's effectiveness not only in Iran but also potentially globally for other domains that require testing and validation of the HTS program. Finally, the present findings can guide policy-makers to develop the required standards and guidelines for implementing theory- and evidence-based HTS cessation programs and interventions.

## Conclusions

The present research supports the effectiveness of the training intervention based on the IM framework in HTS cessation. Statistically significant differences were found in the mean scores of intervention group before and after the training. A significantly lower rate of HTS and a higher rate of cessation were observed in the intervention group compared with the control. Finally, it can be concluded that developing an effective intervention in complicated behaviors such as HTS is demanding but practical. It seems that IM can, through an integrated, systematic, theory-based, or participation-based interventional program, be effective in all steps of program design to reduce or cease the rate of HTS.

## Supplementary Information

**Additional file 1**. Education and training content.

## Data Availability

The datasets analyzed during the current study are available from the Corresponding author upon reasonable request.

## Abbreviations

HTS: Hookah tobacco smoking
IM: Intervention Mapping
