# Supplementary material for: Using intervention mapping for hookah smoking cessation: a quasi-experimental evaluation
Source: Addict Sci Clin Pract. 2022 Mar 14;17:18. doi: 10.1186/s13722-022-00287-5 (PMC8919552; doi:10.1186/s13722-022-00287-5)
Supplement: Supplementary file 1 — Additional file 1. Education and training content. [file 13722_2022_287_MOESM1_ESM.docx]

**Supplementary file 1. Education and training content**

| **Personal level** | | |
| --- | --- | --- |
| **Timing** | **Title** | **Activities** |
| 1^st^ week, 3 hours | Familiarity with HTS adverse effects/ familiarity with temptations | In this session, women were familiarized with the adverse effects and tempting factors of HTS with help of the instructor through lecture, brain-storming, discussion and active participation. |
| 2^nd^ week, 3 hours | How to stay away from temptations | In this session, women were familiarized with how to stay away from the tempting HTS conditions with help of the instructor through lecture, active participatory discussions using the role model. |
| 3^rd^ week, 3 hours | Significance and essentiality of HTS cessation | In this session, the role model was used to invite people who got in trouble due to HTS. Besides, a movie was shown to display HTS adverse effects and compare women smokers and non-smokers to further highlight the significance and essentiality of HTS cessation in women. |
| 4^th^ week, 3 hours | Barriers to HTS cessation, short- and long-term adverse effects of HTS | In this session, women got to know about the adverse effects of HTS with help of the instructor through active participatory discussions, brain-storming, movie show (“death Symphony”), observation of women smokers’ and non-smokers’ faces, and a video on physicians’ comments shown in mass media on the adverse effects of HTS and its mortality rate and concomitant diseases. Then, they got to know about the benefits of HTS cessation through an educational pamphlet, evaluating the barriers of HTS cessation, and learned about the short-term and long-term adverse effects of HTS. |
| **Interpersonal level** | | |
| 5^th^ week, 2 hours | Significance of HTS cessation among women | In this session, the supporters got to know the role model, and the significance and essentiality of HTS cessation and emotional supports along the cessation pathway. |
| 6^th^ week, 2 hours | Useful cessation strategies among women | In this session, the supporters learned about different useful and effective strategies of limiting HTS inside or outside home with help of the instructor through brain-storming and participatory discussions. |
| 7^th^ week, 2 hours | Significance of emotional and social support of women | In this session, the supporters learned about the significant role of emotional support with the help of the instructor and through lecture, brain-storming and active participatory discussion. |
| **Personal level** | | |
| 8^th^ week, 2 hours | Effective HTS cessation strategies | In this session, women were familiarized with different useful and effective strategies about how to stay away from HTS peers and contaminated areas, with help of the instructor and the role model. |
| 9^th^ week, 2 hours | How to resist HTS temptation | In this session, the women got to know how to resist hookah temptations with the help of instructor and the role model, through guided practice and participatory discussions. |
| 10^th^ week, 3 hours | HS habit cessation | In this session, participatory discussion and brainstorming were used to familiarize women with alternative HTS activities and how to stay away from HTS tempting conditions. |
| 11^th^ week, 3 hours | HS habit cessation | In this session, participatory discussion and brainstorming were used to enlist barriers to HS cessation. Then, suggestions were made on how to eliminate these barriers through brainstorming. |
| 12^th^ week, 90 minutes | Risks and outcomes of HS | In this session, participatory discussion and brainstorming were used with the help of a physician to familiarize women with the outcomes of HS. |
| 13^th^ week, 90 minutes | How to prevent and react appropriately in the case of recurrence | In this session, participatory discussion and brainstorming were used in the presence of a clinical psychologist to familiarize women with how to prevent and react appropriately to the recurrence of the old habit. |
| 14^th^ week, 90 minutes | How to control external factors | In this session, participatory discussion and brainstorming were used in the presence of a clinical psychologist to familiarize women with how to control external stimuli. |
| 15^th^ week, 90 minutes | Increasing self-efficacy | In this session, participatory discussion and brainstorming were used in the presence of a clinical psychologist to familiarize women with how to increase self-efficacy. |
| 16^th^ week, 90 minutes | Motivating cessation | In this session, the clinical psychologist familiarized women with the physical and social benefits of HS cessation and helped further motivate women to cease HS. |
| 17^th^ week, 90 minutes | How to resist HS temptation | In this session, the clinical psychologist familiarized women with how to resist HS temptations. |
